# Integrated Transcriptomic and Metabolomic Analyses Uncover the Molecular Mechanisms Underlying Drought Tolerance in *Isodon suzhouensis*

**DOI:** 10.3390/genes17080936

**Published:** 2026-08-11

**Authors:** Fawang Liu, Lei Pan

**Affiliations:** 1 School of Biological and Food Engineering, Suzhou University, Suzhou 234000, China; pl456_789@sina.com; 2 Anhui Engineering Laboratory for Medicinal and Food Homologous Natural Resources Exploration, Hefei Normal University, Hefei 230601, China; 3 Engineering Research Center for Development and High Value Utilization of Genuine Medicinal Materials in North Anhui Province, Suzhou 234000, China

**Keywords:** *I. suzhouensis* (Wangzaozi), drought stress, transcriptome, metabolome

## Abstract

Background/Objectives: This study aims to reveal the physiological and molecular regulatory mechanisms of the genuine medicinal herb *I. suzhouensis* K. F. Zhai, Z. B. Han & S. B. Zhou (Wangzaozi) of Anhui province in response to drought stress, and clarify the regulatory patterns of drought adversity on the accumulation of its medicinal active ingredients. Methods: Mild natural drought treatment was applied to *I. suzhouensis*. Combined with Illumina high-throughput transcriptome sequencing and HPLC-MS/MS targeted metabolomics detection, this study jointly deciphered the dynamic changes in gene expression and metabolite accumulation of *I. suzhouensis* under drought. Key drought-responsive metabolic pathways, core regulatory genes and marker metabolites were screened. Results: A total of 56,823 high-quality unigenes were obtained via transcriptome sequencing, among which 23,580 differentially expressed genes (DEGs) were identified. Functional enrichment analysis revealed that DEGs were predominantly enriched in pathways, including plant hormone signal transduction, phenylpropanoid biosynthesis, flavonoid biosynthesis and photosynthesis. A total of 4171 metabolites were qualitatively and quantitatively characterized via metabolomics, and 1632 differentially expressed metabolites (DEMs) were screened, mainly enriched in phenylpropanoid biosynthesis, tyrosine metabolism, flavone and flavonol biosynthesis pathways. Physiological measurements of antioxidant indices demonstrated that the activities of SOD and POD increased by approximately 2-fold, while PAL activity rose by 1.55-fold, and chlorophyll content decreased significantly. Multi-omics joint analysis indicated that mild drought stress modulates the expression of genes involved in phenylpropanoid, flavonoid and diterpenoid biosynthetic pathways, alters antioxidant enzyme activities, and coordinately regulates the formation of drought tolerance and the accumulation of bioactive compounds in *I. suzhouensis*. Conclusions: This study systematically elucidates the drought response mechanism of *I. suzhouensis* cultivated in northern Anhui province. It provides theoretical evidence and candidate core responsive gene resources for standardized cultivation of *I. suzhouensis* and precise regulation of medicinal quality in drought-prone production areas.

## 1. Introduction

*I. suzhouensis* K. F. Zhai, Z. B. Han & S. B. Zhou, a perennial herb or subshrub medicinal plant belonging to the *Lamiaceae* family, exerts therapeutic effects of clearing heat and detoxifying, activating blood circulation to relieve pain, and dissipating masses and nodules [1]. Clinically, it is widely used for the treatment of sore throat, abdominal masses, and various tumors. *I. suzhouensis*, locally named “Wangzaozi” in Suzhou City, China, is a homologous medicinal and edible genuine herb endemic to northern Anhui province. The genus *Isodon* comprises approximately 150 species, whose pharmacological activities have been extensively investigated over decades [2,3,4]. Suzhou-grown *I. suzhouensis* (Wangzaozi) is rich in flavonoids and diterpenoids, with potent anti-inflammatory, detoxifying, analgesic, hepatoprotective and gastroprotective properties, as well as strong immunomodulatory activity. It is hailed as a “natural botanical antibiotic” in traditional herbal medicine. Early pharmacological records documented its compatibility in preparations for treating lung abscess, pulmonary tuberculosis, osteomyelitis and other disorders [3].

The core medicinal material basis of *I. suzhouensis* (Wangzaozi) consists of antioxidant secondary metabolites, including diterpenoids, flavonoids and phenolic acids [5]. The contents of these bioactive constituents in medicinal plants are highly susceptible to environmental fluctuations [6]. Wild populations of *I. suzhouensis* (Wangzaozi) are primarily distributed in rocky crevices, sloped barren and water-deficient mountain habitats, conferring inherent drought resistance. Nevertheless, persistent drought and extreme water shortage severely restrict field growth, yield stability and medicinal quality consistency, and even lead to plant withering and resource reduction [5,7].

Global warming has intensified drought events in recent years, rendering drought stress the primary abiotic stress limiting standardized large-scale cultivation and quality improvement in *I. suzhouensis* (Wangzaozi). Existing research on *I. suzhouensis* (Wangzaozi) predominantly focuses on chemical isolation, purification and pharmacological validation, while studies on the molecular mechanisms underlying stress responses remain scarce [3,8]. In particular, multi-layered correlation analyses integrating transcriptional regulation, metabolic remodeling and physiological responses under drought are lacking, which hinders comprehensive understanding of drought-tolerant molecular pathways and impedes scientific support for quality regulation and germplasm improvement in arid planting regions.

Transcriptome sequencing comprehensively captures genome-wide transcriptional alterations under drought and enables efficient excavation of key stress-responsive genes and regulatory pathways. LC-MS-based metabolomics accurately identifies diverse secondary metabolites and quantifies their dynamic accumulation, directly reflecting physiological metabolic changes in plants. Integrated transcriptome-metabolome profiling has become a core technical approach to dissect stress response mechanisms, resolve secondary metabolic regulatory networks and mine functional genes in medicinal plants, and has been widely adopted in abiotic stress research across numerous medicinal herb species [9,10,11,12,13,14,15,16]. In our previous study, we performed integrated analysis of genes and metabolites across different tissues of *I. suzhouensis* using transcriptomic and metabolomic approaches. Two functional genes involved in diterpene synthase were identified and cloned. Gene-overexpression assays in *Arabidopsis thaliana* revealed altered accumulation of diterpenoids such as gibberellins in transgenic Arabidopsis. These findings facilitate further dissection of the biosynthetic pathway of terpenoids in *I. suzhouensis* [17].

In this study, *I. suzhouensis* (Wangzaozi) from northern Anhui was subjected to mild natural drought treatment. Antioxidant physiological indices were measured, combined with transcriptome and targeted metabolome profiling to systematically analyze transcriptional variation, metabolite accumulation and physiological adaptation characteristics under drought. Core drought-responsive pathways, pivotal regulatory genes and marker metabolites were excavated to clarify the correlative regulatory mechanism linking drought tolerance formation and the accumulation of primary antioxidant bioactive compounds. This research aims to provide solid theoretical foundations and technical support for drought-resistant germplasm innovation, high-yield and high-quality cultivation in drought-prone regions, and molecular regulation of genuine medicinal quality of *I. suzhouensis* (Wangzaozi).

## 2. Materials and Methods

### 2.1. Plant Materials and Drought Stress Treatment

One-year-old seedlings of *I. suzhouensis* (Wangzaozi) were collected from commercial cultivation bases in northern Anhui province. Seedlings were transplanted into a composite substrate consisting of humus soil, perlite and vermiculite at a volume ratio of 3:1:1. Cultivation conditions were maintained at 25 °C, photosynthetic photon flux density of 12,000 lx, photoperiod of 14 h light/10 h dark, and relative air humidity of 65–70%.

Mild natural drought stress was imposed for 2 weeks; the control group was irrigated every three days. Light, temperature and air humidity were kept consistent throughout the treatment period. Leaf tissues were harvested after two weeks of treatment, immediately snap-frozen in liquid nitrogen, and preserved at −80 °C in an ultra-low-temperature freezer for subsequent physiological measurement, transcriptome sequencing and metabolomic detection.

### 2.2. Determination of Physiological Indices

Fresh leaf samples from control and drought-stressed groups were collected with three biological replicates per group for measurements of superoxide dismutase (SOD), peroxidase (POD), phenylalanine ammonia-lyase (PAL) activities and total chlorophyll content. Commercial assay kits (Solarbio, Beijing, China) were utilized for SOD, POD and PAL activity quantification [11]. SOD activity was determined via NBT spectrophotometry: xanthine oxidase generates superoxide anions to reduce NBT into blue formazan precipitate; SOD eliminates superoxide radicals to suppress color development, and absorbance at 560 nm was recorded to calculate enzyme activity via inhibition rate. POD activity was measured by guaiacol colorimetric assay: H_2_O_2_ served as substrate, and POD catalyzes guaiacol oxidation into brown derivatives; the rising absorbance rate at 470 nm was used to quantify POD activity. PAL activity was detected using UV spectrophotometry: PAL catalyzes L-phenylalanine conversion into *trans*-cinnamic acid, which exhibits characteristic UV absorption at 290 nm; PAL activity was calculated based on absorbance variation at 290 nm. All enzyme activities were standardized as U g^−1^ fresh weight (FW). Chlorophyll content was quantified via 80% acetone extraction-spectrophotometry [18]. Fresh leaf fragments were soaked in acetone in the dark until complete decolorization, followed by volume fixation. Absorbance values at 663 nm and 645 nm were measured, and chlorophyll concentrations were calculated using the Arnon equations: $C_{a}=12.72A_{663}-2.59A_{645}$; $C_{b}=22.88A_{645}-4.67A_{663}$; Total chlorophyll:$\;C_{t}=C_{a}+C_{b}$.

### 2.3. Transcriptome Sequencing and Bioinformatic Analysis

Total RNA was extracted from leaf tissues using the TRIzol reagent. RNA integrity was verified via 1% agarose gel electrophoresis, and RNA purity and concentration were quantified using an Agilent 2100 Bioanalyzer (Agilent Technologies, Santa Clara, CA, USA). Qualified RNA samples were enriched for mRNA, fragmented, reverse-transcribed, end-repaired, tailed with poly-A, ligated with sequencing adapters and amplified by PCR to construct cDNA libraries. Libraries were strictly quality-controlled via Qubit fluorometric quantification and agarose electrophoresis, and then subjected to paired-end sequencing on the Illumina NovaSeq 6000 platform (Illumina, San Diego, CA, USA, accessed on 12 July 2025).

Raw sequencing reads were quality-controlled using FastQC to filter low-quality reads, adapter sequences and redundant sequences, yielding clean high-quality data. Trinity software (v2.15.1, accessed on 20 July 2025) was applied for *de novo* assembly of clean reads to generate high-quality unigenes, followed by sequence redundancy removal via CD-HIT. All unigenes were aligned against six public databases (NR, GO, KEGG, Swiss-Prot, Pfam, eggNOG) for gene functional annotation with an E-value cutoff of 1 × 10^−5^.

DESeq2 software (v1.46.0, accessed on 20 July 2025 ) was used to screen differentially expressed genes (DEGs) between drought and control groups with thresholds of |log_2_FC| ≥ 1 and adjusted *p*-value < 0.05. GO functional enrichment and KEGG pathway enrichment analyses were performed on DEGs, with significantly enriched pathways defined by adjusted *p*-value < 0.05 [8].

### 2.4. Metabolome Profiling and Data Analysis

Fifty milligrams of pulverized leaf samples were mixed with 1 mL pre-cooled 70% methanol aqueous solution, vortexed for 3 min, ultrasonicated in an ice bath for 30 min, and centrifuged at 12,000 r min^−1^ for 15 min at 4 °C. The supernatant was filtered through a 0.22 μm organic membrane and transferred into injection vials. Quality control (QC) samples were prepared to evaluate detection stability. Metabolite separation and quantification were performed on an UPLC-MS/MS equipped with an ACQUITY UPLC HSS T3 column (2.1 mm × 100 mm, 1.8 μm, Waters, Milford, MA, USA, accessed on 2 November 2025). Mobile phase A consisted of 0.1% formic acid in water, and mobile phase B was 0.1% formic acid in acetonitrile with gradient elution at a flow rate of 0.3 mL min^−1^, column temperature of 40 °C and injection volume of 2 μL. The electrospray ionization (ESI) source was operated in dual positive/negative ion mode with spray voltage of 550 V, ion source temperature of 500 °C, and scanning mass range of *m*/*z* 100–1500.

Raw metabolomic data were preprocessed using Progenesis QI software (v2.4, accessed on 15 November 2025) for peak alignment, retention time correction, peak extraction, integration and normalization. Metabolite identification was conducted against METLIN and HMDB databases, and quantitative analysis was performed via multiple reaction monitoring (MRM). Multivariate statistical analyses, including principal component analysis (PCA) and orthogonal partial least squares discriminant analysis (OPLS-DA), were executed in SIMCA14.1 to distinguish metabolic profiles across groups. Differentially expressed metabolites (DEMs) were screened with thresholds of variable importance in projection (VIP) ≥ 1 and *p* < 0.05. DEMs were further annotated to KEGG pathways and subjected to expression clustering analysis to characterize metabolite accumulation patterns [11].

### 2.5. Integrated Transcriptome-Metabolome Analysis and Gene-Metabolite Interaction Network Construction

Pearson correlation analysis was performed between DEGs and DEMs to calculate the correlation coefficient r and corresponding *p*-values. Significant gene-metabolite pairs were filtered with |r| ≥ 0.8 and *p* < 0.05. Combined with KEGG pathway mapping, co-expressed DEGs and DEMs were mapped to shared metabolic pathways. Cytoscape software (v3.10.4, accessed on 4 April 2026) was utilized to visualize gene-metabolite regulatory interaction networks under drought stress, systematically resolve synergistic regulatory mechanisms of core pathways and identify pivotal regulatory nodes.

## 3. Results

### 3.1. Effects of Drought Stress on Physiological Characteristics of I. suzhouensis (Wangzaozi)

**Figure 1:**
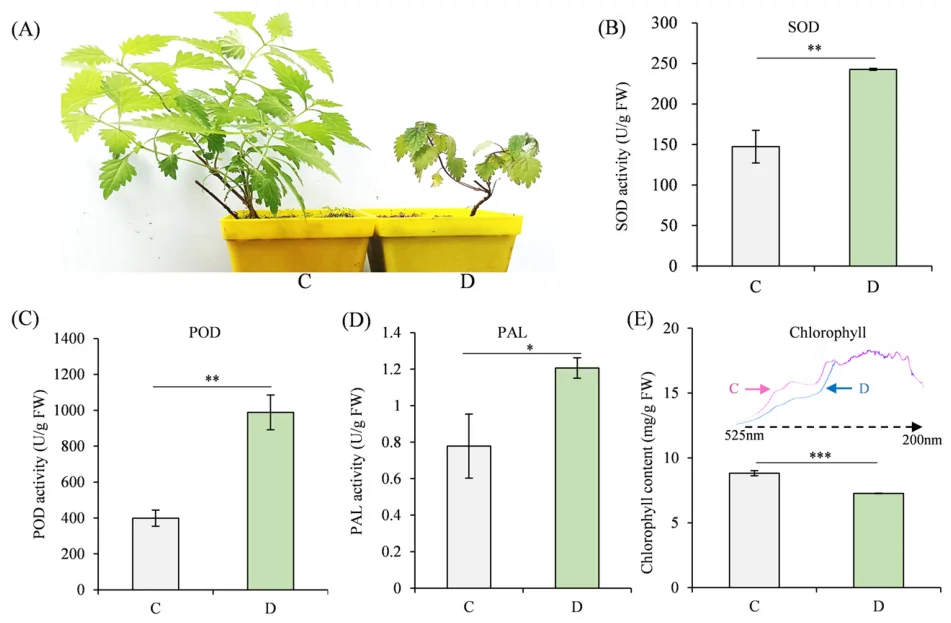
Morphological and physiological traits of *I. suzhouensis* (Wangzaozi) under drought stress. (**A**) Morphological phenotypes of drought-stressed plants; (**B**) SOD activity; (**C**) POD activity; (**D**) PAL activity; (**E**) total chlorophyll content under control and drought treatments. Bars represent mean ± standard deviation (SD) from three biological replicates. C, control; D, drought treated; *, *p* < 0.05; **, *p* < 0.01; ***, *p* < 0.001.

Compared with well-watered control plants, drought-treated *I. suzhouensis* (Wangzaozi) exhibited leaf wilting, yellowing and slight curling without severe irreversible damage to whole plants (Figure 1A). Activities of antioxidant enzymes SOD and POD increased significantly under mild drought. SOD activity rose from 147.41 U g^−1^ FW in controls to 242.68 U g^−1^ FW under drought, representing a 1.65-fold elevation (Figure 1B). POD activity increased from 399.21 U g^−1^ FW to 988.87 U g^−1^ FW, a 2.48-fold increase (Figure 1C). Drought stress induces reactive oxygen species (ROS) accumulation in leaf tissues, and *I. suzhouensis* (Wangzaozi) counteracts oxidative damage by upregulating antioxidant enzyme activities to enhance ROS scavenging capacity. PAL activity increased from 0.78 U g^−1^ FW to 1.21 U g^−1^ FW (1.55-fold higher under drought), indicating that drought modulates PAL activity to stimulate downstream synthesis of total phenols and flavonoids for enhanced antioxidant defense (Figure 1D). Total chlorophyll content decreased markedly from 8.81 mg g^−1^ FW in controls to 7.27 mg g^−1^ FW under drought (Figure 1E). Drought impairs thylakoid membrane structure of chloroplasts, accelerating chlorophyll degradation and inhibiting *de novo* pigment biosynthesis, which substantially weakens light capture efficiency, suppresses photosynthetic carbon fixation and reduces photosynthate accumulation. Chlorophyll attenuation is a typical physiological marker of drought-induced damage, directly reflecting the severity of photosynthetic apparatus injury.

### 3.2. Transcriptome Assembly Quality and Gene Functional Annotation

**Figure 2:**
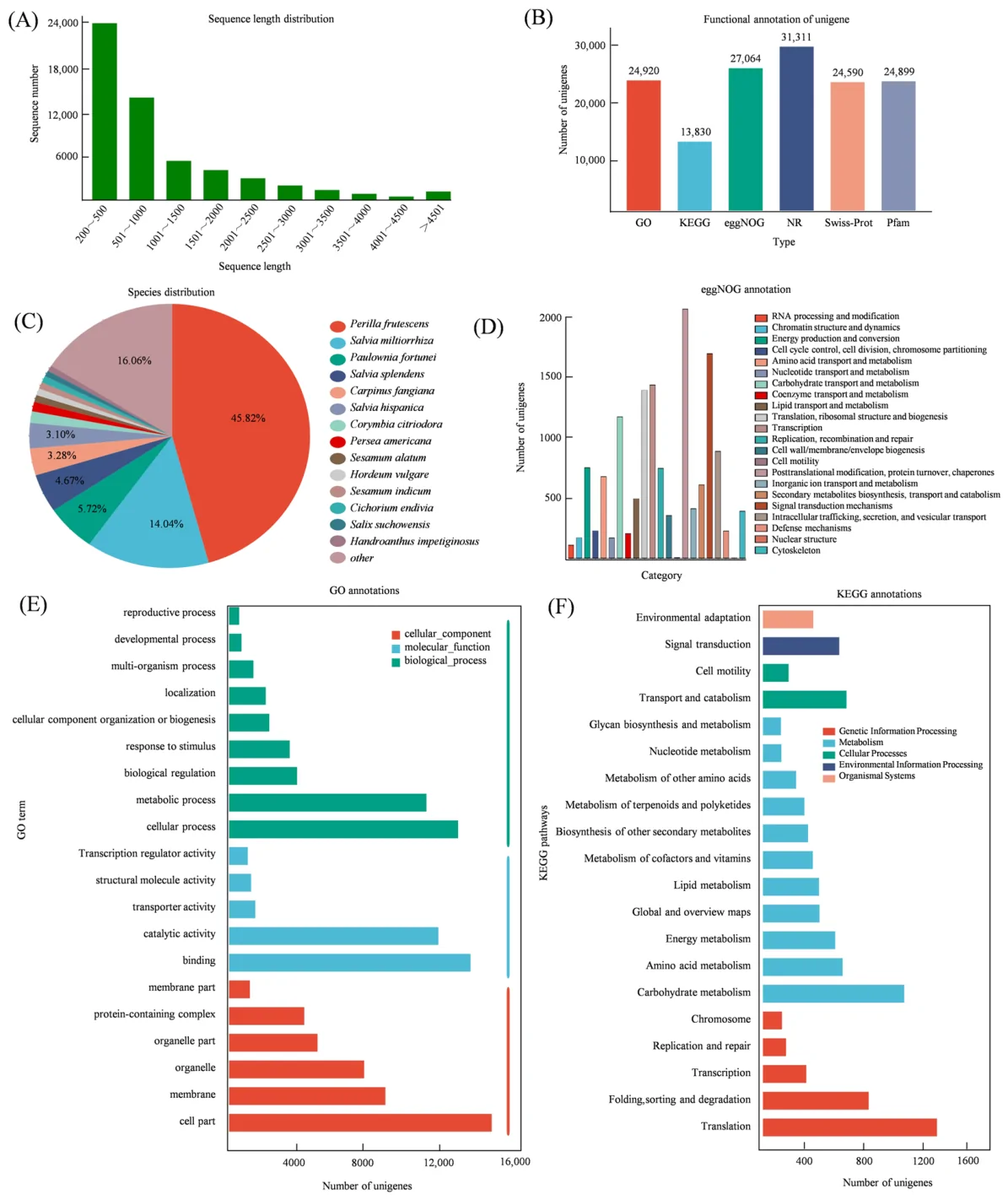
Transcriptome assembly and functional annotation of unigenes. (**A**) Unigene length distribution; (**B**) statistics of unigene functional annotations across six databases; (**C**) species distribution of homologous annotated unigenes; (**D**) eggNOG functional classification; (**E**) GO level-2 classification; (**F**) KEGG level-2 pathway classification.

High-throughput sequencing was performed on six samples, generating a total of 42.23 Gb of clean data, with each sample yielding over 5.88 Gb of clean reads. The Q30 base percentage exceeded 95.42% with uniform GC content distribution. *De novo* assembly of clean reads via Trinity produced 56,823 unigenes and 113,780 transcripts with an average N50 length of 1991 bp. Sequence length distribution revealed that the 200~500 bp interval contained the largest number of unigenes (23,782), followed by the 501~1000 bp interval (13,856). The count of sequences longer than 1000 bp declined sharply, with 17% of total unigenes exceeding 2000 bp in length. The overall length distribution complied with standard quality characteristics of plant *de novo* transcriptome assembly, demonstrating favorable continuity and integrity (Figure 2A).

All Unigenes were aligned against six public databases for homologous functional annotation. The NR database achieved the highest annotation rate, matching 31,311 unigenes (56.04%), followed by eggNOG (27,064, 48.44%), GO (24,920, 44.6%), Pfam (24,899, 44.01%), Swiss-Prot (24,590, 44.01%) and KEGG (13,830, 24.75%). Cumulatively, 88.2% of unigenes obtained functional annotations in at least one database, indicating comprehensive annotation coverage (Figure 2B).

Species homology analysis based on the NR database showed that 14,480 unigenes (45.82%) shared the highest homology with *Perilla frutescens*, followed by 4436 unigenes (14.04%) homologous to *Salvia miltiorrhiza*. Other closely related genera, including *Paulownia*, *Salvia splendens* and *Catharanthus roseus*, each accounted for less than 3%, while 16.06% (5076) sequences were classified as other species. The species distribution of homologous unigenes was consistent with the phylogenetic affinity of *Lamiaceae* species, verifying the taxonomic reliability and conservation of transcriptomic data (Figure 2C).

eggNOG functional annotation revealed that posttranslational modification, protein turnover and chaperones represented the most abundant category (2083 unigenes), followed by signal transduction mechanisms (1571), transcription (1449) and translation, ribosomal structure and biogenesis (1408). Categories including cell motility and nuclear structure were annotated with fewer than 100 unigenes, demonstrating that assembled genes were predominantly enriched in fundamental cellular maintenance and signal regulatory modules (Figure 2D).

GO classification results indicated catalytic activity (12,193 unigenes) and binding (14,056 unigenes) dominated the molecular function category; cell part (15,288 unigenes) and membrane (9114 unigenes) were the most abundant cellular component terms; cellular process (13,335 unigenes) and metabolic process (11,498 unigenes) represented the top biological process entries (Figure 2E).

KEGG pathway annotation mapped all unigenes to five primary and 32 secondary KEGG categories. Metabolism constituted the dominant primary category with 10,780 annotated genes (49.71% of total KEGG annotations), among which carbohydrate metabolism (1220 unigenes), amino acid metabolism (693), energy metabolism (630) and global and overview maps (495) represented the largest secondary subcategories. Genetic information processing ranked second with 3236 annotated genes, including translation (1500), folding, sorting and degradation (916), replication and repair and transcription. Within environmental information processing, 664 unigenes were annotated to signal transduction, while 442 unigenes mapped to environmental adaptation under organismal systems (Figure 2F).

### 3.3. Analysis of Differentially Expressed Genes (DEGs)

**Figure 3:**
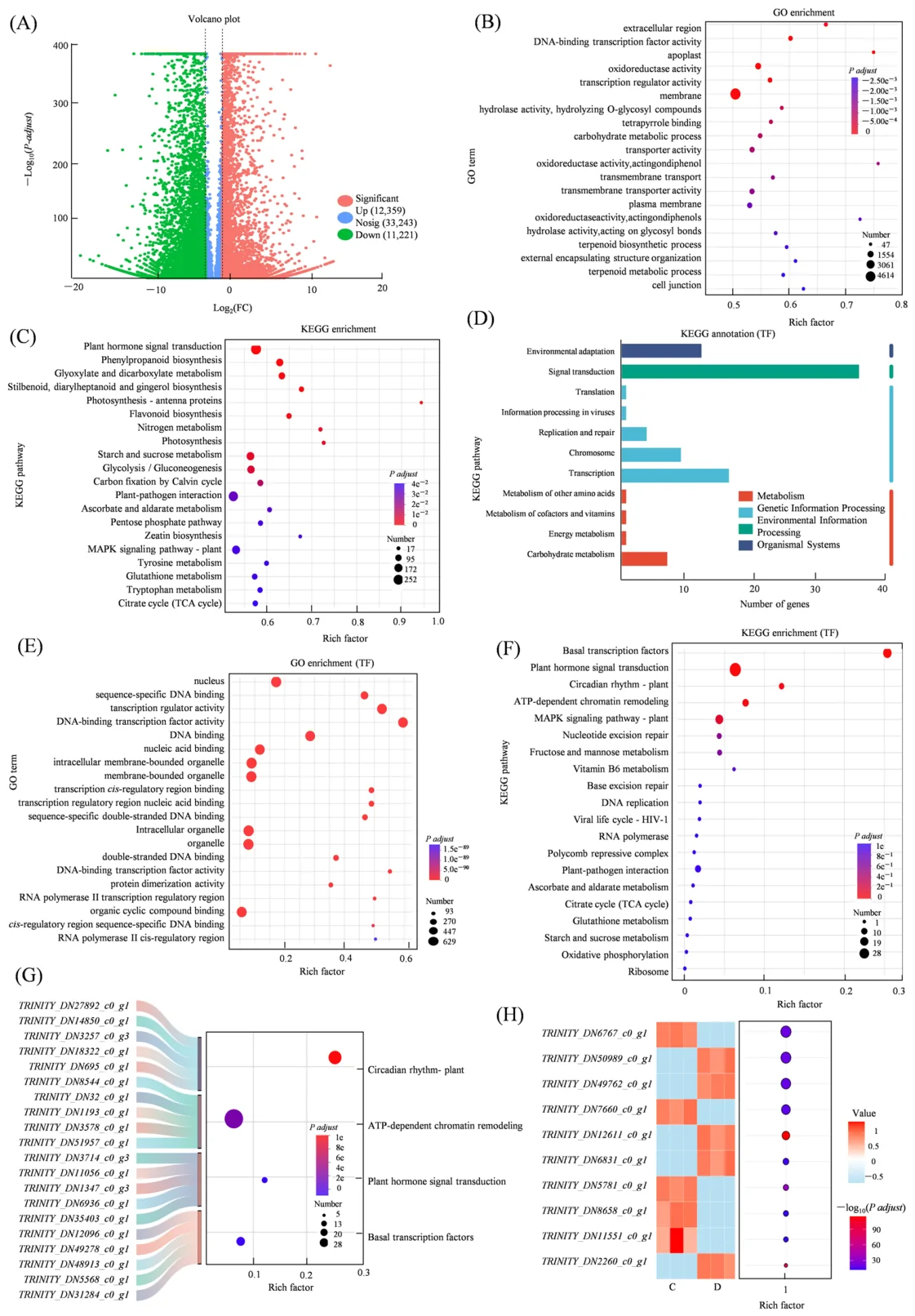
Analysis of differentially expressed genes (DEGs). (**A**) Volcano plot of DEGs; (**B**) GO enrichment of all DEGs; (**C**) KEGG enrichment of DEGs; (**D**) KEGG annotation of differential transcription factors (TFs); (**E**) GO enrichment of differential TFs; (**F**) KEGG enrichment of differential TFs; (**G**) association analysis of TFs with four top enriched KEGG pathways; (**H**) top 10 most significantly differential transcription factors.

A total of 23,580 DEGs were identified under drought stress, consisting of 12,359 upregulated and 11,221 downregulated genes (Figure 3A). GO enrichment analysis of DEGs revealed significant enrichment in apoplast (111 unigenes), oxidoreductase activity acting on diphenols as electron donors with oxygen as acceptor (47 Unigenes), extracellular region (352 unigenes), cell junction (117 unigenes), DNA-binding transcription factor activity (578 unigenes), terpenoid biosynthetic process (202 unigenes) and terpenoid metabolic process (220 unigenes) (Figure 3B).

KEGG enrichment of DEGs identified 20 significantly enriched conserved plant pathways. Photosynthesis-antenna proteins exhibited the most significant enrichment (17 DEGs), followed by photosynthesis (32 DEGs) and nitrogen metabolism (36 DEGs). Other prominent pathways included zeatin biosynthesis (27), flavonoid biosynthesis (65), stilbenoid, diarylheptanoid and gingerol biosynthesis (61), phenylpropanoid biosynthesis (124), glyoxylate and dicarboxylate metabolism (104) and tyrosine metabolism (65). Plant hormone signal transduction harbored the maximum number of DEGs (252), followed by plant-pathogen interaction (211), MAPK signaling pathway (157), starch and sucrose metabolism (145) and glycolysis/gluconeogenesis (134) (Figure 3C).

Differentially expressed transcription factors (TFs) were screened from DEGs for KEGG annotation, with signal transduction containing the largest number of annotated unigenes (35), followed by translation (16), environmental adaptation (12), chromosome (9) and carbohydrate metabolism (7). This suggests that transcription factors coordinate drought responses via signal transduction and secondary metabolic regulation (Figure 3D).

GO enrichment of differential TFs identified DNA-binding transcription factor activity (573 unigenes) as the most enriched term, followed by RNA polymerase II-specific DNA-binding transcription factor activity (134) and transcription regulator activity (576), while organic cyclic compound binding (584) represented the least enriched category (Figure 3E).

KEGG enrichment of differential TFs highlighted four core pathways: basal transcription factors, plant hormone signal transduction, circadian rhythm and ATP-dependent chromatin remodeling. Plant hormone signal transduction contained the largest number of annotated TFs (28 unigenes). Highly differentially expressed genes within each pathway were identified, including bZIP, bHLH, GRAS, TCP, CONSTANS-like and histone CBF/NF-Y family transcription factors (Figure 3F,G). The top 10 most significantly differentially expressed transcription factors included RAD-like 6, zinc finger ACE1, TFIIB, ERF016, WRKY50, bHLH75 and GATA12 (Figure 3H).

### 3.4. Metabolite Annotation Under Drought Stress

**Figure 4:**
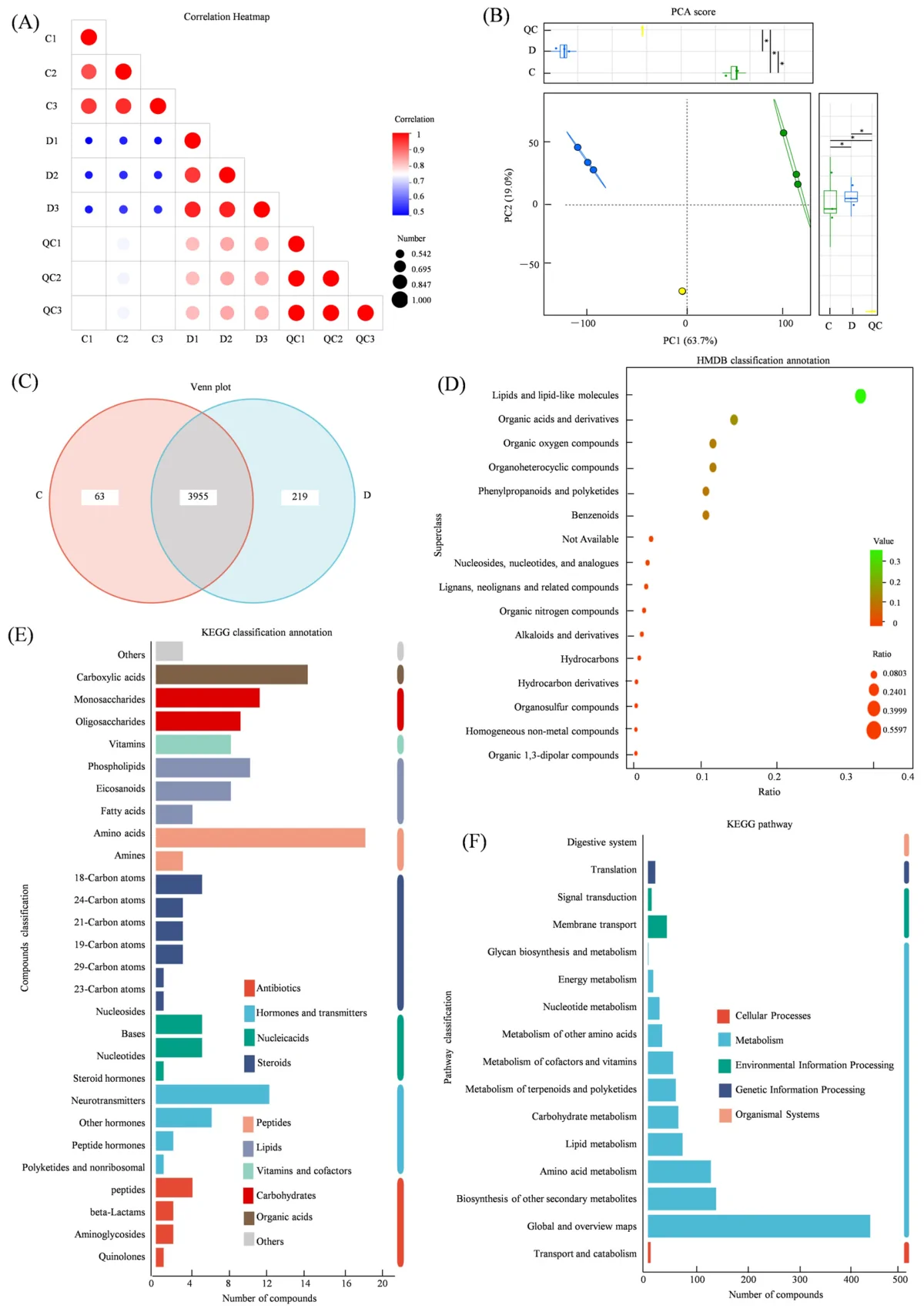
Metabolite annotation profiling of *I. suzhouensis* (Wangzaozi). (**A**) Sample correlation heatmap of metabolomic data; (**B**) PCA of all detected metabolites, * *p* < 0.05; (**C**) Venn diagram of metabolites shared between control and drought groups; (**D**) HMDB superclass classification of annotated metabolites; (**E**) KEGG compound classification; (**F**) KEGG pathway distribution of annotated metabolites.

Non-targeted LC-MS/MS profiling was performed on control and drought-treated leaf samples. Correlation heatmaps showed intra-group correlation coefficients > 0.55 for control replicates and >0.95 for drought replicates. PCA analysis yielded PC1 = 63.7% and PC2 = 19.0%, demonstrating high reproducibility of metabolomics datasets (Figure 4A,B).

A total of 4171 metabolites were detected across six samples, among which 2574 were annotated via HMDB and Lipidblast databases, and 1329 obtained KEGG annotations covering 105 metabolic pathways. Venn diagram analysis indicated 3955 shared metabolites between groups, 63 metabolites exclusively detected in controls and 219 metabolites unique to drought-stressed plants (Figure 4C).

HMDB superclass classification revealed lipids and lipid-like molecules as the most abundant annotated class (809 metabolites, 32.62%). Other major classes with over 100 annotated metabolites included organic acids and derivatives (368, 14.84%), organic oxygen compounds (290, 11.69%), organoheterocyclic compounds (289, 11.65%), phenylpropanoids and polyketides (258, 10.40%) and benzenoids (255, 10.28%) (Figure 4D).

KEGG compound classification prioritized amino acids (22 metabolites), steroid hormones (12), monosaccharides (11), oligosaccharides (9) and eicosanoids (8) (Figure 4E). KEGG pathway distribution showed global and overview maps containing the maximum annotated metabolites (426), followed by biosynthesis of other secondary metabolites (132), amino acid metabolism (122), lipid metabolism (68) and carbohydrate metabolism (60) (Figure 4F).

### 3.5. Identification of Differentially Expressed Metabolites (DEMs)

**Figure 5:**
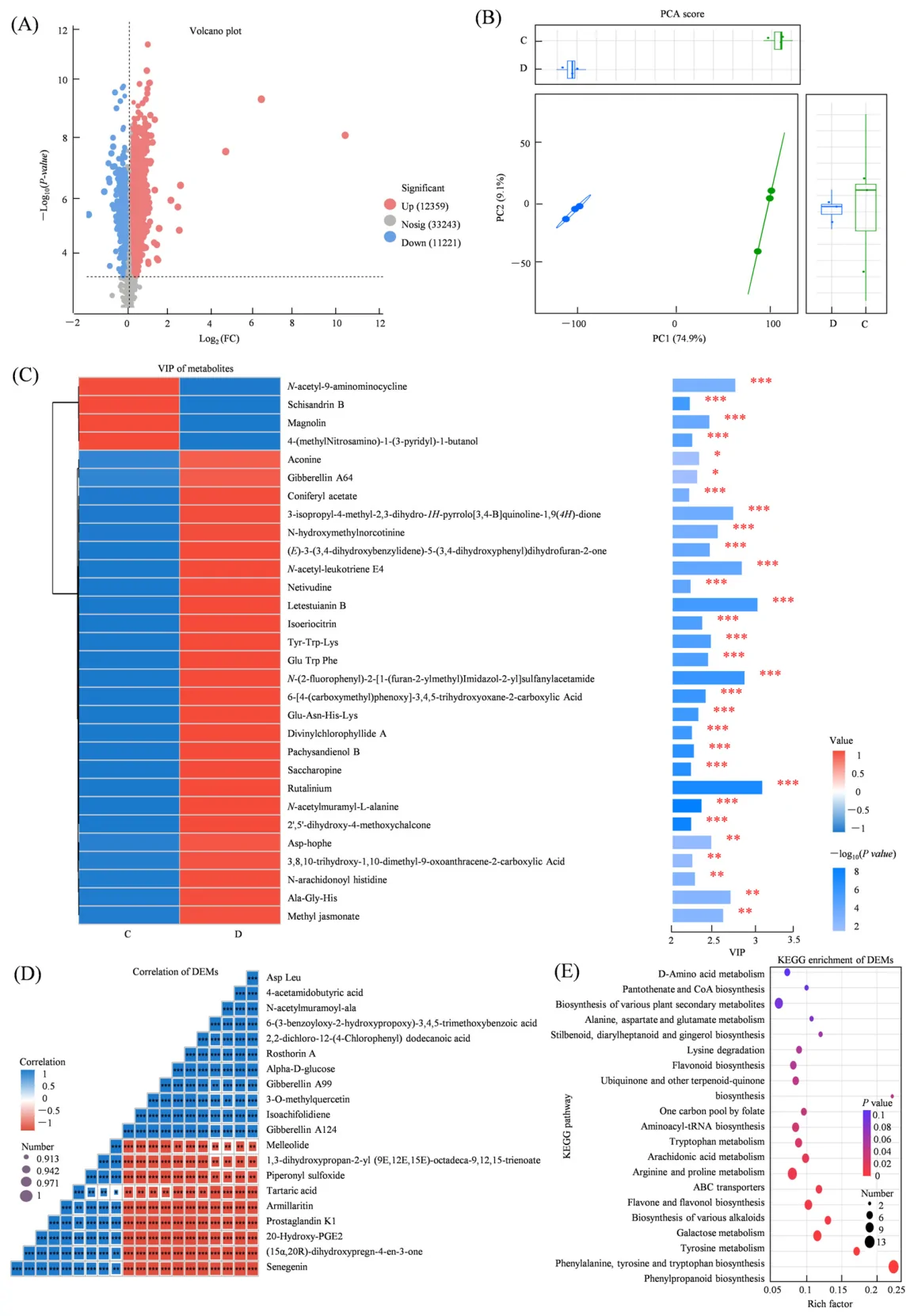
Analysis of differentially expressed metabolites (DEMs). (**A**) Volcano plot of DEMs; (**B**) PCA of DEM profiles; (**C**) heatmap of top variable metabolites; (**D**) correlation matrix of representative metabolites; (**E**) KEGG enrichment bubble plot of DEMs. *, *p* < 0.05; **, *p* < 0.01; ***, *p* < 0.001.

Comparative analysis between drought and control groups identified 1632 DEMs, comprising 1251 upregulated and 381 downregulated metabolites (Figure 5A). PCA of DEMs revealed tight intra-group clustering and clear inter-group separation between control and drought samples (Figure 5B).

Metabolites with the largest fold changes were characterized: *N*-acetyl-9-aminominocycline, magnolin, 4-(methylnitrosamino)-1-(3-pyridyl)-1-butanol and schisandrin B decreased markedly under drought, while rutalinium, letestuianin B, methyl jasmonate, and *N*-hydroxymethylnorcotinine accumulated significantly (Figure 5C). Metabolite correlation analysis identified strong positive correlations between 4-acetamidobutyric acid and multiple phenolic and terpenoid metabolites, as well as a tight correlation between senegenin and triterpenoid and prostaglandin derivatives (Figure 5D).

KEGG enrichment of DEMs identified phenylpropanoid biosynthesis as the most significantly enriched pathway (13 annotated DEMs), followed by phenylalanine, tyrosine and tryptophan biosynthesis (6), tyrosine metabolism (9), galactose metabolism (6), biosynthesis of various alkaloids (9) and flavone and flavonol biosynthesis (6). Additional enriched pathways included arginine and proline metabolism, arachidonic acid metabolism, tryptophan metabolism, ubiquinone and other terpenoid-quinone biosynthesis and flavonoid biosynthesis (Figure 5E).

### 3.6. Integrated Transcriptome and Metabolome Analysis

**Figure 6:**
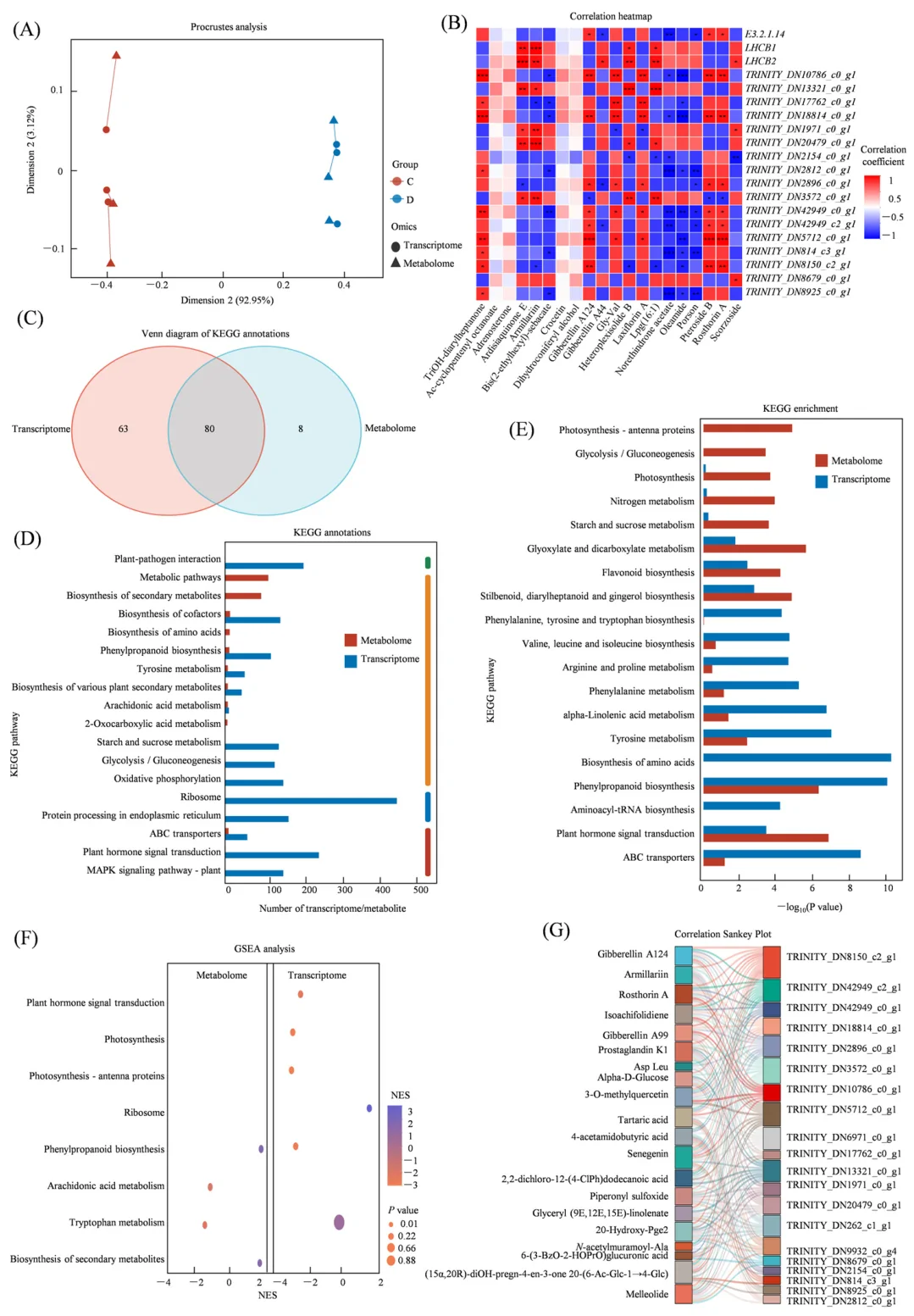
Integrated transcriptome-metabolome multi-omics analysis. (**A**) Procrustes joint PCA of transcriptomic and metabolomic profiles; (**B**) gene-metabolite correlation heatmap; (**C**) Venn diagram of shared KEGG pathways annotated by two omics; (**D**) statistics of gene/metabolite counts in major KEGG pathways; (**E**) bubble plot of KEGG enrichment for transcriptome and metabolome; (**F**) GSEA enrichment results; (**G**) Sankey diagram of top 20 significant gene-metabolite correlation pairs. *, *p* < 0.05; **, *p* < 0.01; ***, *p* < 0.001.

Procrustes joint PCA of transcriptomic and metabolomic datasets demonstrated strong inter-omics correlation and complete separation between control and drought groups, confirming that drought drastically reshapes transcriptional and metabolic landscapes of *I. suzhouensis* (Wangzaozi) (Figure 6A). Pearson correlation analysis identified multiple gene-metabolite pairs with perfect correlation coefficients (r = 1), including armillaritin with *TRINITY_DN20479_c0_g1*, heteroplexisolide B with *TRINITY_DN13321_c0_g1* and gibberellin A124 with *TRINITY_DN5712_c0_g1* (Figure 6B).

Venn analysis of KEGG pathways annotated by transcriptome and metabolome revealed 80 shared pathways, 63 transcriptome-specific pathways and eight metabolome-exclusive pathways. Transcriptome datasets dominated signal transduction and primary metabolic pathways, while metabolomics enriched secondary metabolite biosynthesis pathways (Figure 6C–E). GSEA enrichment confirmed that plant hormone signal transduction, photosynthesis and phenylpropanoid biosynthesis were transcriptionally enriched, whereas phenylpropanoid biosynthesis, arachidonic acid metabolism and secondary metabolite biosynthesis dominated metabolic enrichment (Figure 6F). Sankey diagram of the top 20 significantly correlated gene-metabolite pairs highlighted drought-responsive genes annotated to “response to water deprivation” (GO annotation term: 0009414) linked to gibberellins, armillaritin and rosin diterpenoid metabolites (Figure 6G).

### 3.7. Integrated Analysis of Genes and Metabolites in Phenylpropanoid Biosynthesis (Ko00940) Pathway

**Figure 7:**
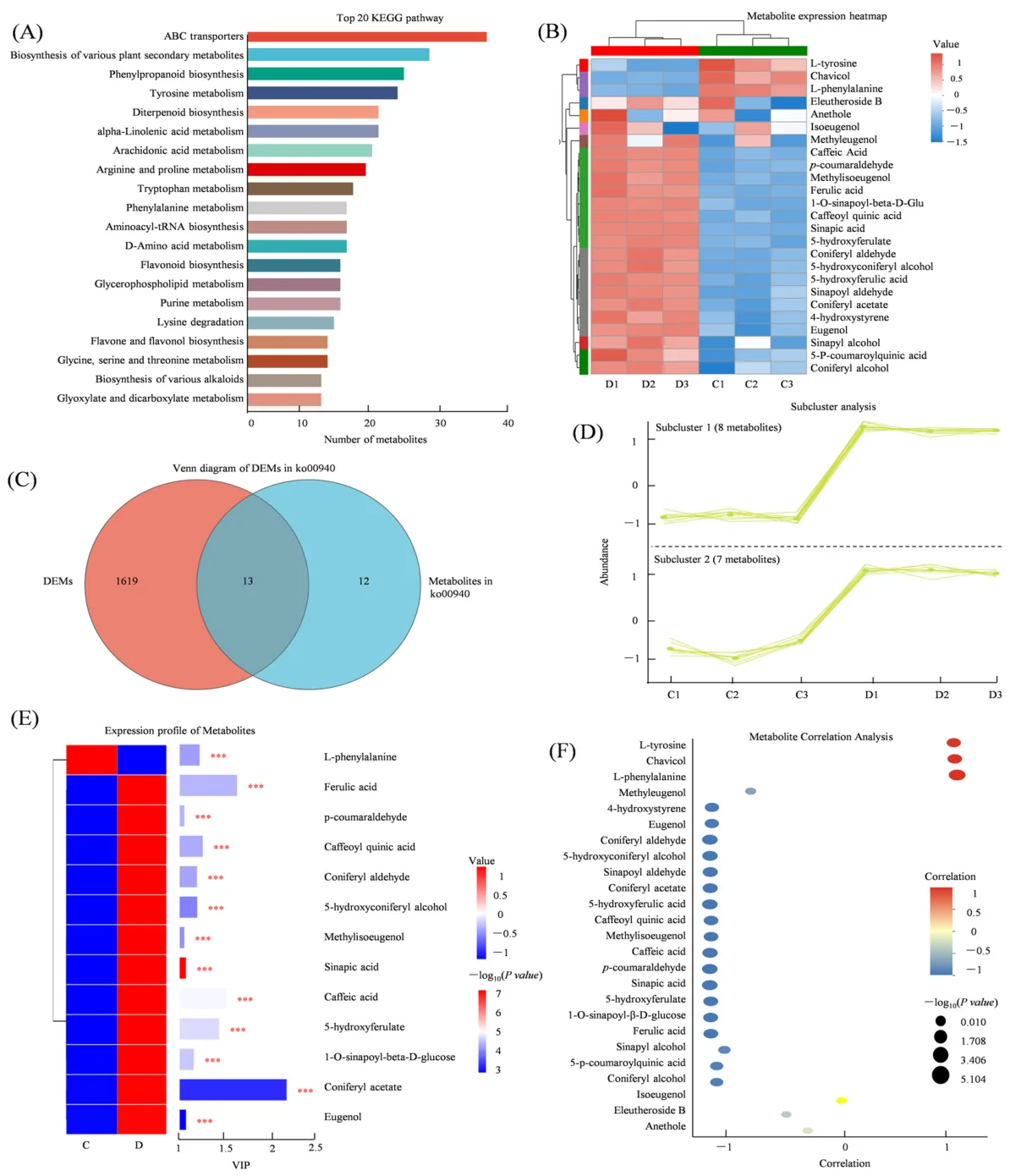
Profiling of metabolites in the phenylpropanoid biosynthesis pathway (Ko00940). (**A**) Ranking of KEGG pathways by annotated metabolite count; (**B**) expression heatmap of 25 phenylpropanoid metabolites; (**C**) Venn diagram of phenylpropanoid DEMs against all detected metabolites; (**D**) subcluster expression trends of 25 metabolites; (**E**) VIP plot of 13 differential phenylpropanoid metabolites; (**F**) correlation matrix of all 25 pathway metabolites. ***, *p* < 0.001.

A total of 25 metabolites were annotated to the phenylpropanoid biosynthesis pathway, 13 of which were identified as DEMs. All metabolites exhibited distinct accumulation patterns between control and drought groups (Figure 7A–C). Hierarchical clustering partitioned metabolites into 10 subclusters; subcluster 1 (eight metabolites) and subcluster 2 (seven metabolites) were significantly upregulated under drought. Among the 13 DEMs, only L-phenylalanine decreased, while 12 phenolic metabolites accumulated drastically, with coniferyl acetate, ferulic acid, caffeic acid, 5-hydroxyferulate and caffeoyl quinic acid showing the largest fold increases (Figure 7D). Correlation analysis revealed a strong positive correlation between L-tyrosine, L-phenylalanine and chavicol, whereas these three precursors displayed significant negative correlation with downstream phenolic derivatives such as coniferyl alcohol and ferulic acid (Figure 7F).

**Figure 8:**
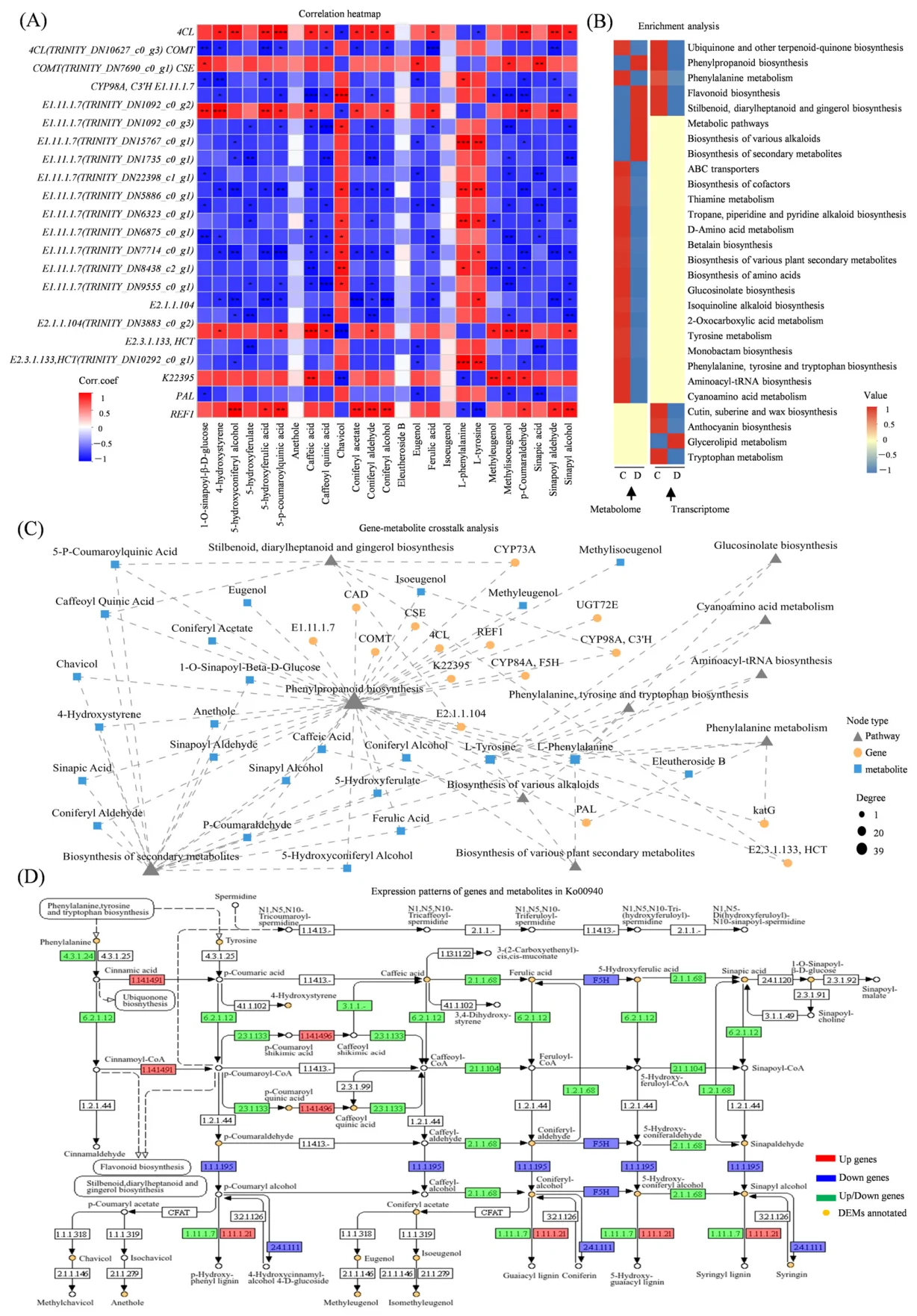
Integrated gene-metabolite analysis of phenylpropanoid biosynthesis pathway. (**A**) Correlation heatmap between core structural genes and pathway metabolites; (**B**) enrichment cross-talk of pathway DEGs with other metabolic modules; (**C**) network of shared genes/metabolites across interconnected pathways; (**D**) co-expression heatmap of DEGs and DEMs within Ko00940. *, *p* < 0.05; **, *p* < 0.01; ***, *p* < 0.001.

Joint analysis of 124 DEGs and 25 pathway metabolites demonstrated consistent transcriptional and metabolic variation trends. Key structural genes including *PAL*, *4CL*, *COMT* and *CYP98A (C3′H)* exhibited positive correlation with caffeic acid, ferulic acid and sinapyl alcohol, while negative correlation with upstream aromatic amino acid precursors (Figure 8A). Cross-pathway crosstalk was observed, as *HCT* and 5-*P*-coumaroylquinic acid simultaneously participated in phenylpropanoid and stilbenoid biosynthesis pathways (Figure 8C). Co-expression heatmaps verified synchronous upregulation of pathway enzymes and antioxidant phenolic metabolites under drought (Figure 8D).

### 3.8. Integrated Analysis of Genes and Metabolites in Tyrosine Metabolism (Ko00350) Pathway

**Figure 9:**
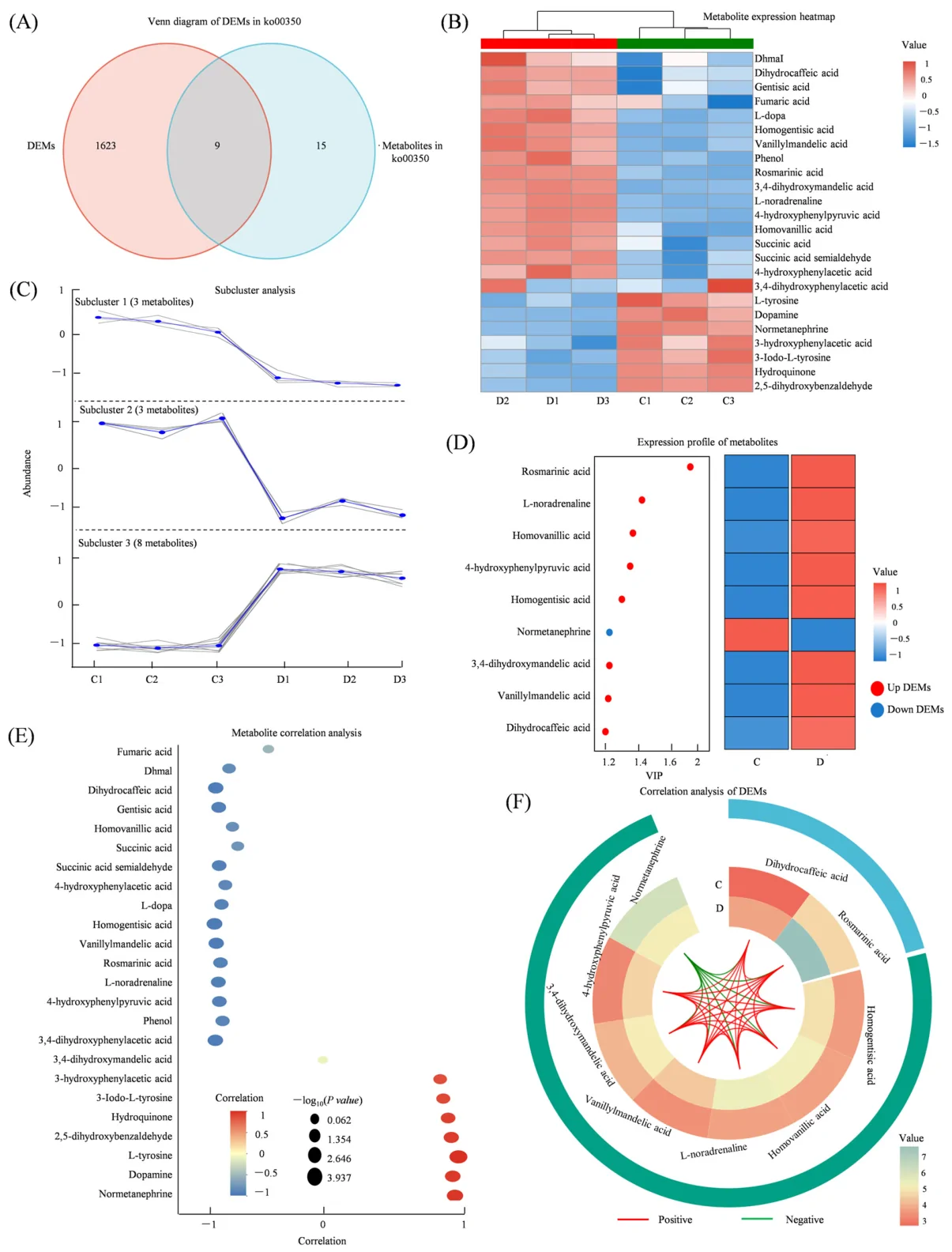
Profiling of metabolites in tyrosine metabolism pathway (Ko00350). (**A**) Venn diagram of tyrosine metabolites against global metabolome; (**B**) expression heatmap of 24 tyrosine-derived metabolites; (**C**) dominant expression subclusters; (**D**) VIP plot of nine differential metabolites; (**E**) correlation matrix of all 24 pathway metabolites; (**F**) correlation network of nine DEMs.

Twenty-four metabolites were mapped to tyrosine metabolism, among which nine were DEMs with distinct accumulation profiles between groups (Figure 9A). Ten expression subclusters were identified; subcluster 1 and 2 metabolites were downregulated, while subcluster 3 metabolites accumulated significantly under drought (Figure 9B,C). Only normetanephrine decreased among nine DEMs, whereas rosmarinic acid, dihydrocaffeic acid and homogentisic acid exhibited dramatic upregulation (Figure 9D). L-tyrosine displayed a strong positive correlation with upstream aromatic precursors and a negative correlation with downstream phenolic antioxidants (Figure 9E), and normetanephrine showed a universal negative correlation with all other eight differential tyrosine-derived metabolites (Figure 9F).

**Figure 10:**
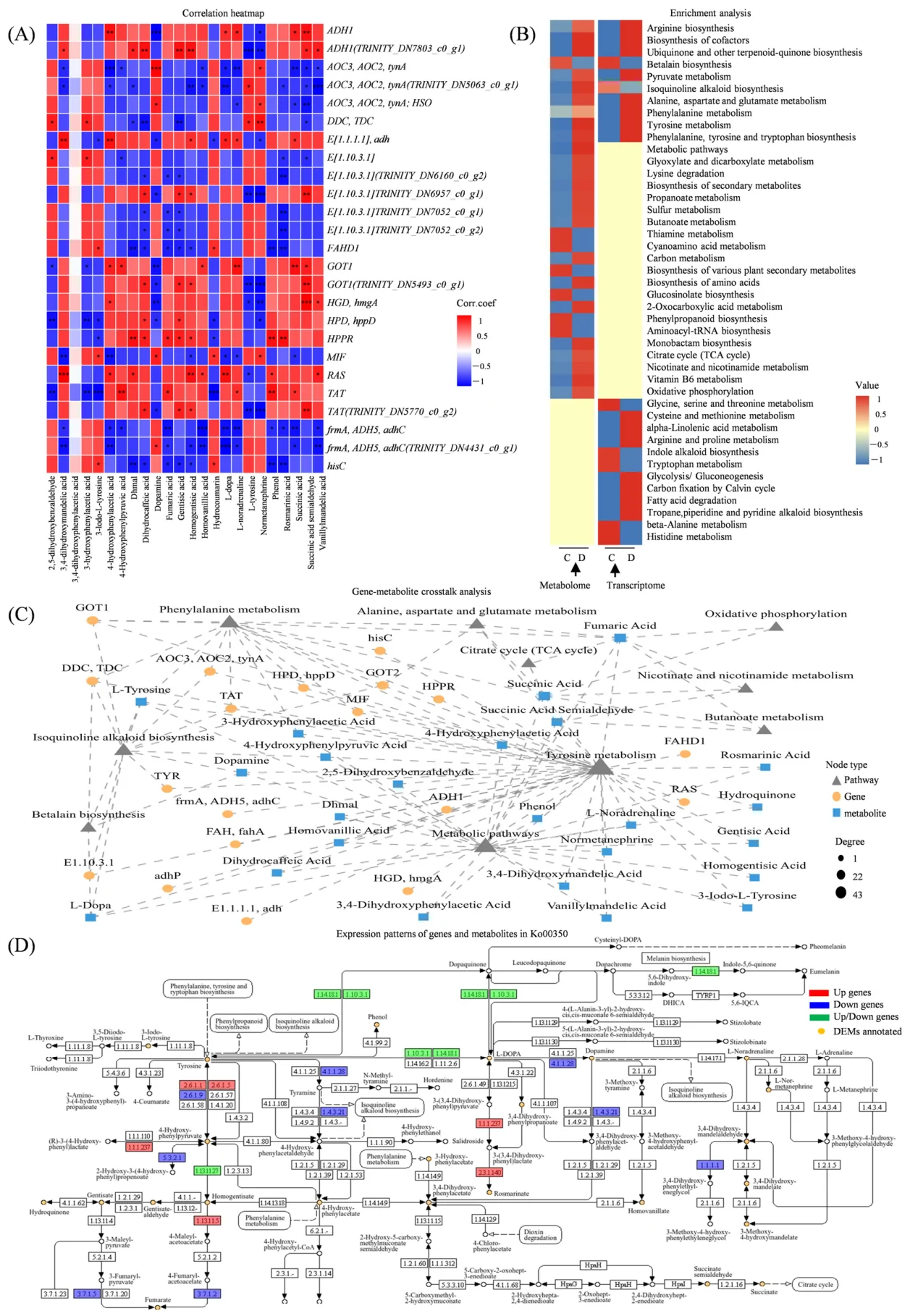
Integrated gene-metabolite analysis of tyrosine metabolism pathway. (**A**) Correlation heatmap of core enzyme genes and pathway metabolites; (**B**) cross-pathway enrichment of tyrosine DEGs; (**C**) inter-pathway crosstalk network; (**D**) co-expression clustering of DEGs and DEMs in ko00350. * *p* < 0.05; ** *p* < 0.01; *** *p* < 0.001.

Joint profiling of 54 DEGs and 24 tyrosine metabolites identified pivotal catalytic genes *TAT*, *HPD* and *ADH1* with strong positive correlation with downstream antioxidant metabolites and negative correlation with L-tyrosine and dopamine (Figure 10A). Cross-pathway crosstalk was detected for *GOT1* and fumaric acid linking tyrosine metabolism to phenylalanine metabolism, the TCA cycle and isoquinoline alkaloid biosynthesis (Figure 10C). Co-expression heatmaps confirmed coordinated transcriptional and metabolic reprogramming of the tyrosine pathway under drought (Figure 10D).

### 3.9. Integrated Analysis of Genes and Metabolites in Diterpenoid Biosynthesis (Ko00904) Pathway

**Figure 11:**
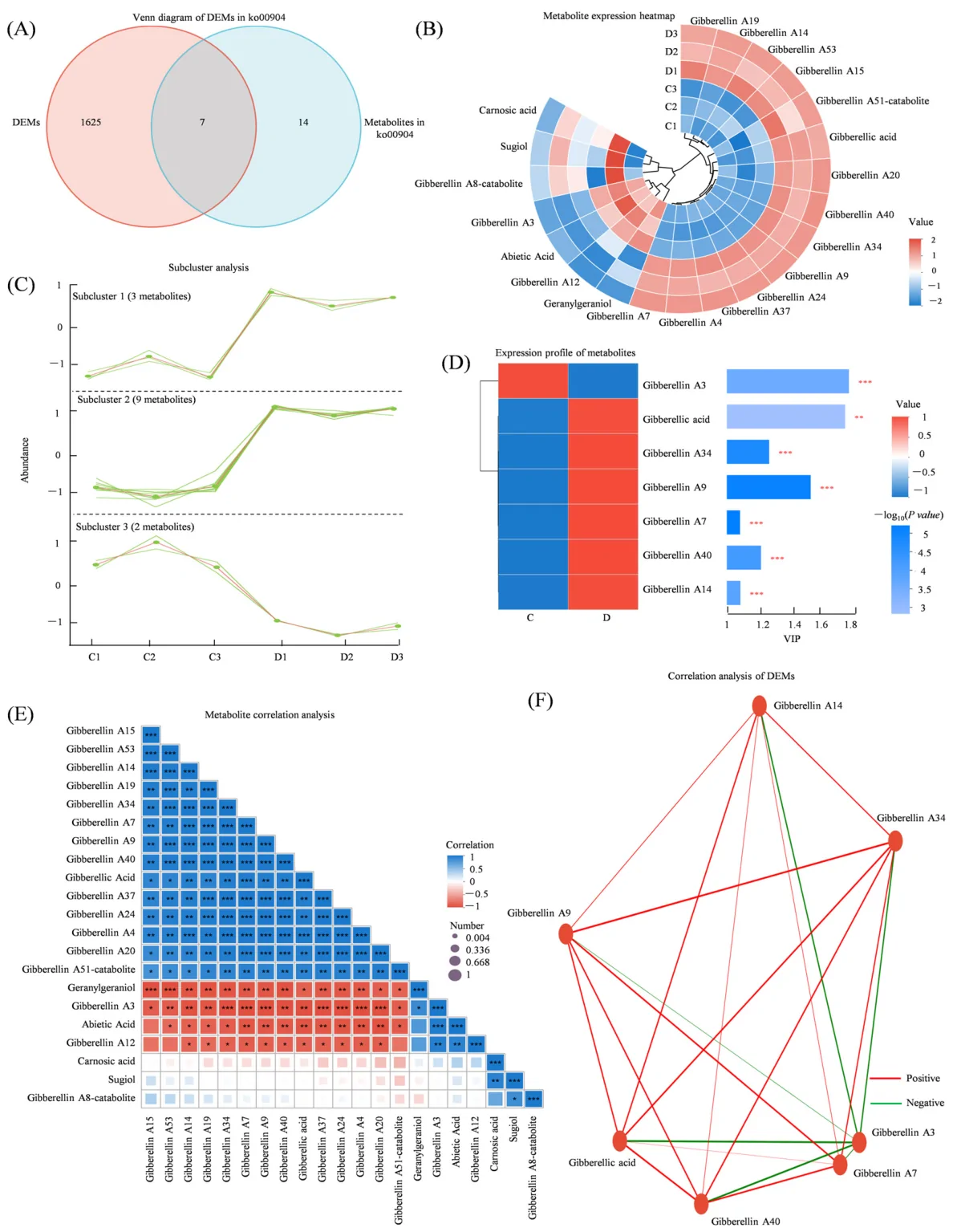
Profiling of metabolites in diterpenoid biosynthesis pathway (Ko00904). (**A**) Venn diagram of diterpenoid metabolites; (**B**) expression heatmap of 21 diterpenoids; (**C**) major expression subclusters; (**D**) VIP plot of seven differential diterpenoid metabolites; (**E**) correlation matrix of all 21 pathway metabolites; (**F**) correlation network of seven DEMs. *, *p* < 0.05; **, *p* < 0.01; ***, *p* < 0.001.

Twenty-one metabolites were annotated to diterpenoid biosynthesis, with seven classified as DEMs (Figure 11A). Ten expression subclusters were resolved; subcluster 1 and 2 diterpenoids (predominantly gibberellin derivatives) were significantly upregulated under drought, while subcluster 3 metabolites declined (Figure 11B,C). Among seven DEMs, only Gibberellin A3 decreased, while Gibberellic acid, Gibberellin A7, A9, A14, A34 and A40 accumulated markedly (Figure 11D). Carnosic acid and sugiol exhibited positive correlation with gibberellin catabolites but negative correlation with growth-suppressive Gibberellin A3 (Figure 11E), and Gibberellin A3 showed strong negative correlation with all six upregulated gibberellin DEMs (Figure 11F).

**Figure 12:**
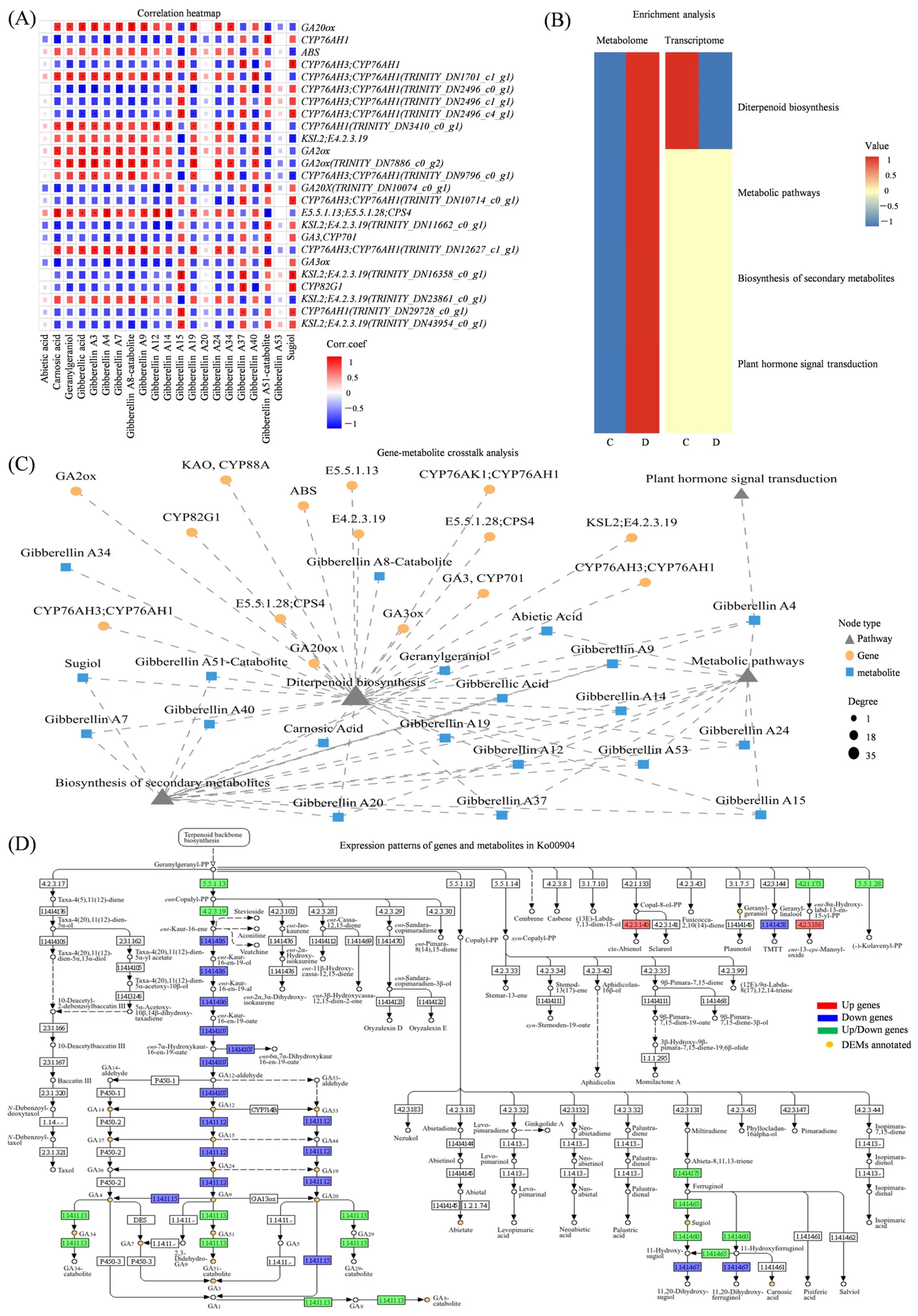
Integrated gene-metabolite analysis of diterpenoid biosynthesis pathway. (**A**) Correlation heatmap of diterpenoid synthase genes and pathway metabolites; (**B**) functional enrichment of diterpenoid DEGs; (**C**) cross-pathway metabolic crosstalk; (**D**) co-expression clustering of DEGs and DEMs in Ko00904. *, *p* < 0.05; **, *p* < 0.01; ***, *p* < 0.001.

Fifty-four DEGs were annotated exclusively to diterpenoid biosynthesis. Key enzyme genes including *CYP76AH3/CYP76AH1*, *GA2ox* and *KSL* displayed distinct correlation patterns with gibberellin and abietic acid derivatives (Figure 12A,B). Secondary metabolic crosstalk connected diterpenoid biosynthesis to global secondary metabolite pathways (Figure 12C). Co-expression heatmaps validated consistent transcriptional activation of diterpenoid biosynthetic enzymes and medicinal terpenoid metabolites under mild drought (Figure 12D).

### 3.10. Integrated Analysis of Genes and Metabolites in Flavonoid Biosynthesis (Ko00941), Flavone and Flavonol Biosynthesis (Ko00944) Pathways

**Figure 13:**
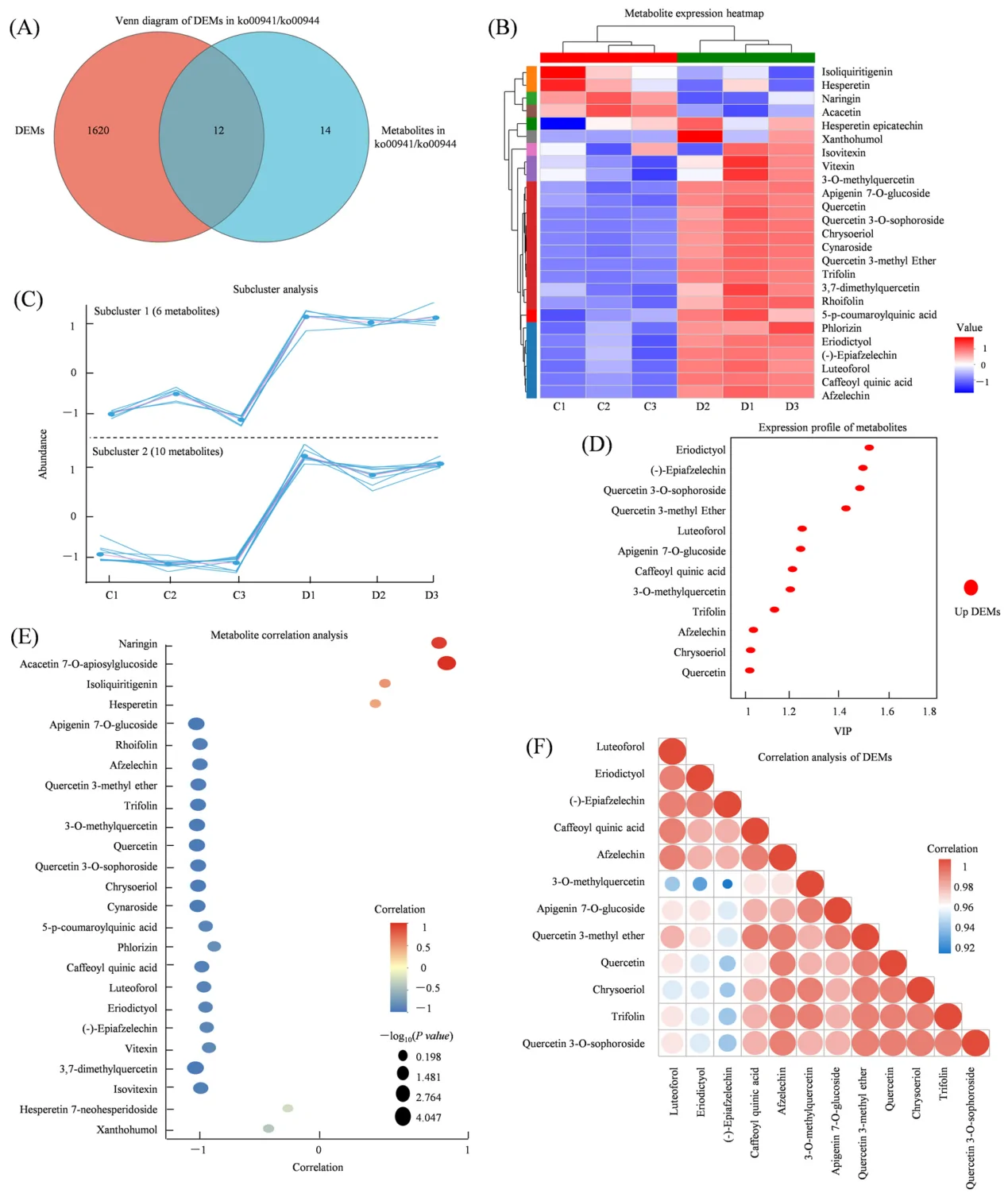
Profiling of metabolites in flavonoid and flavone and flavonol biosynthesis pathways (Ko00941, Ko00944). (**A**) Venn diagram of 26 flavonoid metabolites against global metabolome; (**B**) expression heatmap of all pathway metabolites; (**C**) two dominant upregulated subclusters; (**D**) VIP plot of 12 differential flavonoid metabolites; (**E**) correlation matrix of all 26 metabolites; (**F**) correlation network of 12 DEMs.

Twenty-six metabolites were jointly annotated to flavonoid and flavone and flavonol biosynthetic pathways, with 12 identified as DEMs (Figure 13A). Ten expression subclusters were generated; subcluster 1 (6 metabolites) and subcluster 2 (10 metabolites) represented flavonoid derivatives significantly upregulated under drought (Figure 13B,C). All 12 DEMs accumulated under drought, with Eriodictyol, (-)-Epiafzelechin, quercetin glycosides and apigenin glucoside exhibiting the largest fold changes (Figure 13D). Differential flavonoid metabolites displayed universal positive inter-correlation, consistent with coordinated pathway activation under drought (Figure 13E,F).

**Figure 14:**
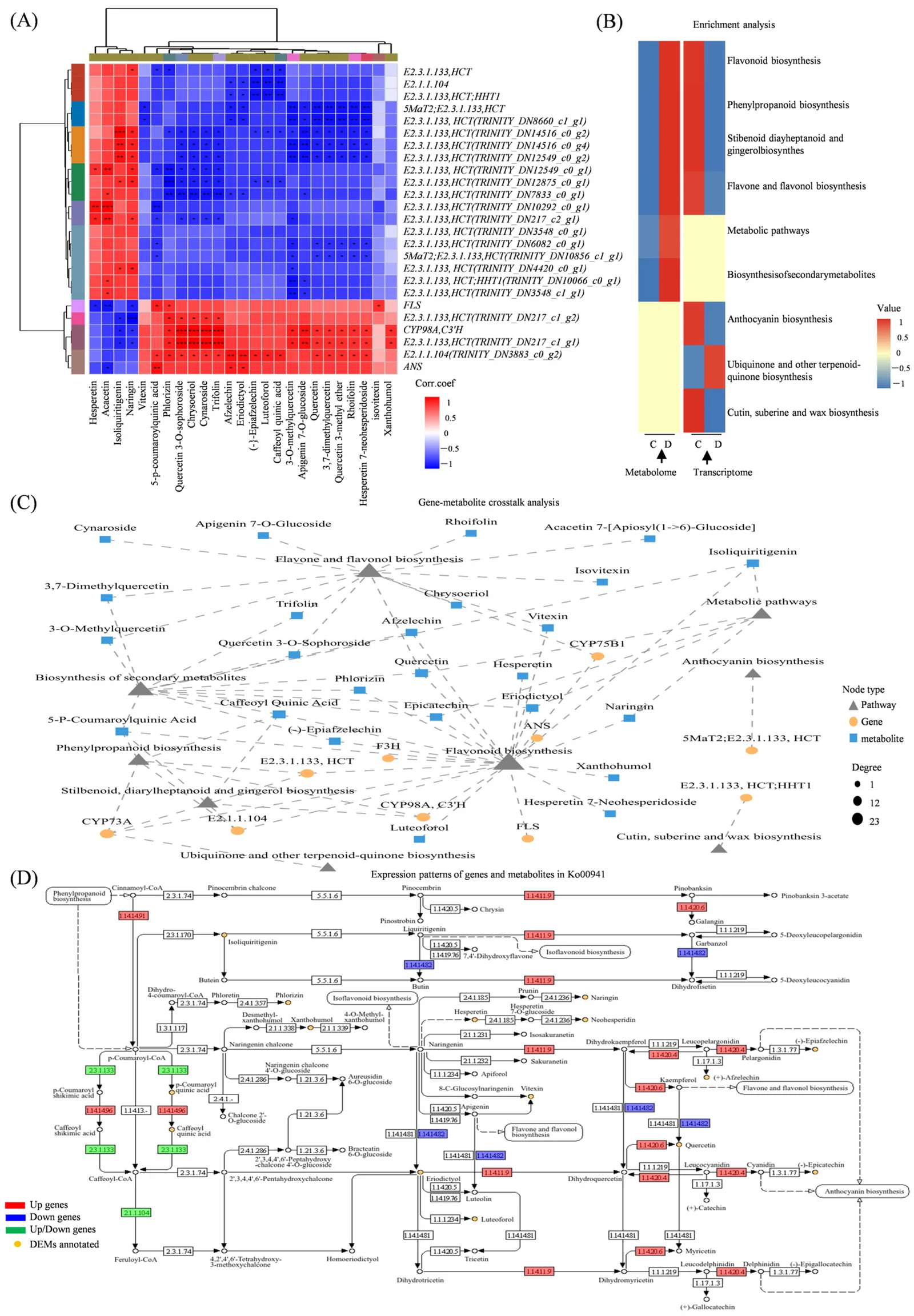
Integrated gene-metabolite analysis of flavonoid and flavonol biosynthetic pathways. (**A**) Correlation heatmap of core flavonoid synthase genes and pathway metabolites; (**B**) cross-pathway enrichment of flavonoid DEGs; (**C**) inter-pathway metabolic crosstalk network; (**D**) co-expression heatmap of DEGs and DEMs within Ko00941/Ko00944. *, *p* < 0.05; **, *p* < 0.01; ***, *p* < 0.001.

Sixty-five DEGs were mapped to flavonoid and flavonol biosynthesis, including structural genes *HCT*, *FLS*, *C3′H* and *ANS* with distinct correlation patterns to upstream flavanone precursors and downstream antioxidant flavonol end-products (Figure 14A,B). Extensive pathway crosstalk linked flavonoid biosynthesis to phenylpropanoid, stilbenoid and anthocyanin metabolic networks (Figure 14C). Co-expression clustering confirmed synchronous induction of flavonoid biosynthetic genes and medicinal flavonoid metabolites under mild drought stress (Figure 14D).

## 4. Discussion

This study integrated physiological biochemistry, high-throughput transcriptome and UPLC-MS/MS targeted metabolomics to systematically dissect physiological damage, transcriptional reprogramming and coordinated metabolic regulation of northern Anhui genuine herb *I. suzhouensis* (Wangzaozi) under mild drought stress. The correlative molecular linkage among drought adversity, drought tolerance and medicinal bioactive compound accumulation was preliminarily evaluated, filling critical research gaps regarding drought-responsive molecular mechanisms of *I. suzhouensis* (Wangzaozi) and providing theoretical guidance for quality-oriented cultivation in drought-prone planting regions.

Physiological phenotypic responses revealed that mild drought induced leaf chlorosis and wilting alongside significant chlorophyll reduction, consistent with canonical drought symptoms in terrestrial plants [19]. Drought disrupts thylakoid membrane integrity, simultaneously inhibiting chlorophyll biosynthesis and accelerating pigment degradation, thereby suppressing photosynthetic carbon assimilation and restraining plant vegetative growth [14]. Concurrently, leaf SOD and POD activities nearly doubled, while PAL activity increased by 1.55-fold, forming a complete antioxidant defense cascade. SOD and POD synergistically eliminate drought-triggered ROS to mitigate membrane lipid peroxidation, constituting the basal physiological barrier for drought tolerance. PAL acts as the rate-limiting enzyme of phenylpropanoid biosynthesis; its upregulation redirects carbon flux from primary vegetative growth toward phenolic acid and flavonoid antioxidant secondary metabolism, achieving dual physiological functions of oxidative stress resistance and medicinal compound accumulation [15,16]. This physiological coordination pattern resembles drought response characteristics of other *Lamiaceae* medicinal herbs such as *Perilla frutescens* and *S. miltiorrhiza*, yet the concurrent activation of diterpenoid biosynthetic pathways confers species-specific physiological-metabolic regulatory features unique to *I. suzhouensis* (Wangzaozi) [20,21].

Transcriptomic data uncovered upstream transcriptional signaling and regulatory networks governing drought perception. *De novo* assembly generated 56,823 high-quality unigenes, with 23,580 total DEGs (12,359 upregulated, 11,221 downregulated). DEGs were significantly enriched in plant hormone signal transduction, photosynthesis, phenylpropanoid, flavonoid and diterpenoid biosynthetic pathways. Global downregulation of photosynthetic genes corresponded perfectly with reduced chlorophyll and suppressed photosynthetic efficiency. Plant hormone signal transduction contained the largest number of DEGs; numerous drought-responsive transcription factors, including bZIP, WRKY, ERF and bHLH, were differentially expressed, transmitting drought signals via ABA and gibberellin signaling cascades as upstream master switches to activate downstream secondary metabolic structural genes [22]. Species homology annotation confirmed closest phylogenetic affinity with *P. frutescens*, validating the authenticity and reliability of *de novo* transcriptome assembly [23]. Hierarchical regulation between transcription factors and secondary metabolic structural genes forms the core transcriptional module for drought signal perception, transduction and adaptive response in *I. suzhouensis* (Wangzaozi).

Metabolomic profiling visualized dynamic accumulation patterns of medicinal constituents under drought. A total of 4171 metabolites were identified, with 1632 DEMs dominated by 1251 upregulated compounds and only minor downregulation of primary metabolites. This demonstrates that mild drought reshapes metabolic resource allocation, prioritizing secondary metabolite biosynthesis. Four core drought-responsive metabolic pathways were identified: phenylpropanoid, tyrosine, flavonoid and diterpenoid biosynthesis. Within phenylpropanoid metabolism, only the precursor L-phenylalanine slightly decreased, while antioxidant phenolic acids, including caffeic acid, ferulic acid and chlorogenic acid, accumulated massively [24]. Rosmarinic acid and related bioactive derivatives were markedly elevated in the tyrosine pathway [25]. All 12 differential flavonoid and flavonol metabolites were upregulated under drought, with substantial increases in quercetin and luteolin glycosides. Most gibberellin diterpenoids accumulated in the diterpenoid pathway except Gibberellin A3. These four categories of metabolites represent the primary therapeutic compounds responsible for the anti-inflammatory, hepatoprotective and detoxifying efficacy of *I. suzhouensis* (Wangzaozi), confirming that moderate drought stress significantly enhances the intrinsic medicinal quality of this herb.

Integrated transcriptome-metabolome analysis further elucidated multi-layered coordinated regulatory mechanisms linking transcription and metabolism. Eighty shared KEGG pathways were annotated across both omics datasets, and expression trends of DEGs and DEMs within phenylpropanoid, flavonoid and diterpenoid pathways exhibited high consistency. Key structural enzymes including 4CL, CYP98A, FLS and GA2ox displayed extremely significant positive correlation with marker medicinal metabolites, collectively constructing a gene-metabolite interaction regulatory network activated under drought [26]. Drought signals are transmitted hierarchically via transcription factors to upregulate rate-limiting enzymes of secondary metabolic pathways, driving massive biosynthesis of antioxidant and pharmacologically active metabolites.

Nevertheless, this study bears certain limitations. Only a single mild drought treatment was implemented without gradient severe drought comparisons, preventing determination of the critical drought threshold balancing biomass yield and medicinal quality [27]. Experiments were conducted under controlled potted indoor conditions, failing to simulate field compound stresses, including soil infertility and high temperature, which restricts direct field application of the present findings. Future research will establish gradient drought treatments to screen optimal water management regimes for high-quality cultivation, conduct multi-year multi-site field trials, and formulate standardized high-yield high-quality cultivation protocols for *I. suzhouensis* (Wangzaozi) in arid production zones. Collectively, this study systematically resolves physiological and molecular drought response mechanisms of northern Anhui *I. suzhouensis* (Wangzaozi), verifies that mild drought simultaneously enhances drought tolerance and medicinal secondary metabolite accumulation, and excavates candidate genes regulating drought resistance and medicinal quality. It provides solid theoretical foundations and genetic resources for drought-resistant germplasm breeding, standardized cultivation and directional quality regulation of *I. suzhouensis* (Wangzaozi) in drought-prone regions.

## 5. Conclusions

In this study, the northern Anhui genuine medicinal herb *I. suzhouensis* (Wangzaozi) was subjected to mild drought stress treatment, followed by integrated physiological measurement, transcriptome and targeted metabolome profiling. Drought significantly elevated the activities of antioxidant enzymes SOD, POD and PAL while reducing leaf chlorophyll content. A total of 23,580 DEGs and 1632 DEMs were identified, with phenylpropanoid biosynthesis, tyrosine metabolism, flavonoid biosynthesis and diterpenoid biosynthesis constituting the core drought-responsive metabolic pathways. Mild drought modulates the transcriptional expression of key pathway enzymes to promote massive accumulation of flavonoids, phenolic acids and diterpenoid medicinal active compounds, forming a coordinated regulatory model coupling drought tolerance and bioactive constituent biosynthesis. This research systematically reveals the molecular drought response mechanism of *I. suzhouensis* (Wangzaozi) and excavates candidate genes governing drought resistance and medicinal quality, providing theoretical evidence and genetic resources for drought-resistant germplasm breeding, standardized cultivation and directional quality control of *I. suzhouensis* (Wangzaozi) in drought-affected production areas.

## Data Availability

The transcriptomic data supporting the findings of this study are openly available under NCBI reference number PRJNA12327553.

## Abbreviations

DEGs	Differentially expressed genes
DEMs	Differentially expressed metabolites
SOD	Superoxide dismutase
POD	Peroxidase
PAL	Phenylalanine ammonia-lyase
FW	Fresh weight
ADH1	Alcohol dehydrogenase 1
C3′H	Coumaroyl-CoA 3′-hydroxylase
COMT	Caffeic acid O-methyltransferase
CYP	Cytochrome P450
GA	Gibberellin
GA2ox	Gibberellin 2-oxidase
GSEA	Gene set enrichment analysis
HMDB	Human metabolome database
KEGG	Kyoto encyclopedia of genes and genomes
MAPK	Mitogen-activated protein kinase
NR	Non-redundant protein database
OPLS-DA	Orthogonal partial least squares discriminant analysis
Pfam	Protein family database
ROS	Reactive oxygen species
Swiss-Prot	Swiss-Prot protein database
VIP	Variable importance in projection
eggNOG	Evolutionary genealogy of genes: Non-supervised Orthologous Groups

## References

[1] Ma H.J., Zhai K.F., Han Z.B., Zhou S.H. (2022). *Isodon suzhouensis* (Lamiaceae): A new species from Anhui. J. Suzhou Univ..

[2] Wei M.Q., Chen L., Zhang W.Q., Sun M., Wang J.J., Zhan J.P., Wang C.L., Yan C. (2025). Chemical constituents from *Isodon suzhouensis* and their antitumor activity. Nat. Prod. Res. Dev..

[3] Duan H., Wang W., Li S., Li H., Khan G.J., Ma Y., Liu F.W., Zhai K.F., Hu H.G., Wei Z.J. (2024). The potential mechanism of *Isodon suzhouensis* against COVID-19 via EGFR/TLR4 pathways. Food Sci. Hum. Wellness..

[4] Zhao C.L., Zhou L., Xie W.J., Zhao L.H., Zhang C.Y., He K., Ye J.H., Zhang J.J., Pan L.T., Zhou J. (2022). Bioactive isopimarane and 3,4-seco isopimarane diterpenoids from *Isodon amethystoides*. BMC Chem..

[5] Duan H., Wang G.C., Khan G.J., Su X.H., Guo S.L., Niu Y.M., Cao W.G., Wang W.T., Zhai K.F. (2021). Identification and characterization of potential antioxidant components in *Isodon amethystoides* (Benth.) Hara tea leaves by UPLC-LTQ-Orbitrap-MS. Food Chem. Toxicol..

[6] Kuljarusnont S., Iwakami S., Iwashina T., Tungmunnithum D. (2024). Flavonoids and other phenolic compounds for physiological roles, plant species delimitation, and medical benefits: A promising view. Molecules.

[7] Ji Q.Y., Chen S.M., Cao Y.N., Xiang X.S., Sun Y.Y., Zhang Y., Xing W.B., Wang Y.Y., Yang Q.S. (2025). Prediction of potential habitat of *Isodon amethystoides* in China under climate change based on optimized MaxEnt model. Front. Plant Sci..

[8] Wang W., Li H., Lv J.M., Khan G.J., Duan H., Zhu J., Bao N.N., Zhai K.F., Xue Z.L. (2023). Determination of the anti-oxidative stress mechanism of *Isodon suzhouensis* leaves by employing bioinformatic and novel research technology. ACS Omega.

[9] Jan R., Asaf S., Numan M., Lubna, Kim K.-M. (2021). Plant secondary metabolite biosynthesis and transcriptional regulation in response to biotic and abiotic stress conditions. Agronomy.

[10] Zhou Y., Bai Y.H., Han F.X., Chen X., Wu F.S., Liu Q., Ma W.Z., Zhang Y.Q. (2024). Transcriptome sequencing and metabolome analysis reveal the molecular mechanism of *Salvia miltiorrhiza* in response to drought stress. BMC Plant Biol..

[11] Liu F.W., Xu Y.Y., Pan L., Zhang Y., Ding M.P. (2026). Transcriptome and metabolome based analysis reveals the molecular mechanisms underlying the differences in tanshinone and salvianolic acid content between *Salvia miltiorrhiza* roots and leaves. Genes.

[12] Mao J.W., Liu J.H., Hou Y.Y., Fang X., Cheng X.Q., Tian L., Ma Y., Yin H.B., Chen X.X. (2025). Transcriptome and metabolome profiling of the medicinal plant *Dictamnus dasycarpus* reveal key genes involved in quinoline alkaloids biosynthesis and limonoids biosynthesis. BMC Plant Biol..

[13] Jiao Y.M., Li X., Chen Y., He Q., Xue Y.J., Zhang Z.Q., Shen Q., Wang P.T., Cao L. (2026). Integrated transcriptome and metabolome analysis of the regulation of flavonoid biosynthesis in *Potentilla anserina* L. under different abiotic stress. Ind. Crops Prod..

[14] Li L.X., He L.X.Z., Jia Z.Q., Li Q.X., Li J.L., Yang Q.K., Zheng Y.Q. (2026). Comparative physiological and multi-omics responses reveal divergent drought-response patterns in two *Melilotus* species. Plant Sci..

[15] Patil J.R., Mhatre K.J., Yadav K., Yadav L.S., Srivastava S., Nikalje G.C. (2024). Flavonoids in plant-environment interactions and stress responses. Discov. Plants.

[16] Zhang S.W., Qi X.L., Zhu R.Y., Ye D.D., Shou M.Y., Peng L.L., Qiu M.H., Shi M., Kai G.Y. (2024). Transcriptome analysis of *Salvia miltiorrhiza* under drought stress. Plants.

[17] Liu F.W., Zhai K.F., Xie D.M. (2025). Heterologous overexpression and functional analysis of the *Isodon suzhouensis IsKS1* gene in *Arabidopsis thaliana*. Curr. Issues Mol. Biol..

[18] Porra R.J. (2002). The chequered history of the development and use of simultaneous equations for the accurate determination of chlorophylls a and b. Photosynth. Res..

[19] Yang X.Y., Lu M.Q., Wang Y.F., Wang Y.R., Liu Z.J., Chen S. (2021). Response mechanism of plants to drought stress. Horticulturae.

[20] Yang R.K., Du Z.Y., Qiu T., Sun J., Shen Y.T., Huang L.L. (2021). Discovery and functional characterization of a diverse diterpene synthase family in the medicinal herb *Isodon lophanthoides* var. *gerardiana*. Plant Cell Physiol..

[21] Zhang X.X., Zhu L.L., Xu Z.H., Wang Z.M., Dai L.P. (2025). Characterization and differential expression of DNA methyltransferase and demethylase genes in response to abiotic stress in *Isodon rubescens*. BMC Plant Biol..

[22] Yang X.H., Chen M.H., Liu M.W., Li B.W., Sun Z.C., Lu A.L., Zhang S., Shi X.H., Ren J., Qin X.Z. (2025). ABA-GA antagonism and modular gene networks cooperatively drive acquisition of desiccation tolerance in perilla seeds. Front. Plant Sci..

[23] Fukushima A., Nakamura M., Suzuki H., Saito K., Yamazaki M. (2015). High-throughput sequencing and *de novo* assembly of red and green forms of the *Perilla frutescens* var. *crispa* transcriptome. PLoS ONE.

[24] Sharma A., Shahzad B., Rehman A., Bhardwaj R., Landi M., Zheng B.S. (2019). Response of phenylpropanoid pathway and the role of polyphenols in plants under abiotic stress. Molecules.

[25] Zhou Y., Ma G.Y., Li W.L., Xie L.P., Zhan S.X., Yao X.D., Zuo Z.W., Tian D.Q. (2025). Analysis of volatile metabolome and transcriptome in sweet basil under drought stress. Curr. Issues Mol. Biol..

[26] Kumar M., Kumar Patel M., Kumar N., Bajpai A.B., Siddique K.H.M. (2021). Metabolomics and molecular approaches reveal drought stress tolerance in plants. Int. J. Mol. Sci..

[27] Wang J., Wan X.X., Liu Q.X., Zhang Y., Tian B., Chen C.L., Gu R.H., Wang B., Chen J.Z., Chen L. (2025). Physiological and molecular mechanism analysis of *Cyclocodon lancifolius* seedlings in response to varying degrees of drought stress. BMC Plant Biol..

